# Determinants of Severely Prolonged Emergency Department Stays Among Older Adults: A Retrospective Comparative Analysis

**DOI:** 10.1155/jonm/5173443

**Published:** 2025-10-19

**Authors:** Zhongxiang Wang, Lan Lai, Xiaping Zhang, Yajun Sun

**Affiliations:** ^1^Department of Nursing, The First Affiliated Hospital, Zhejiang University School of Medicine, Hangzhou, Zhejiang, China; ^2^Department of Nursing, Hangzhou Cancer Hospital, Hangzhou, Zhejiang, China

**Keywords:** aged, emergency service, length of stay, multidisciplinary care, risk factors

## Abstract

**Objective:**

Prolonged emergency department (ED) stays are linked to adverse outcomes in older adults. This study examined the clinical characteristics and determinants of severely prolonged ED stays in a large tertiary hospital in China.

**Methods:**

A retrospective cohort study was conducted at a tertiary hospital in Zhejiang Province, including 652 adult patients with ED boarding ≥ 72 h between January 1 and December 31, 2024. Patients were stratified into geriatric (≥ 60 years) and nongeriatric groups for descriptive comparison. Logistic regression analyses were performed to identify independent predictors of severely prolonged stays (> 96 h) among geriatric patients.

**Results:**

Among all included patients, 73.3% were geriatric. Compared with nongeriatric patients, older adults had significantly higher rates of multimorbidity and nutritional risk and were less likely to arrive at night. Multivariate analysis showed that advanced age, nighttime arrival, nutritional risk, and infection were independent risk factors for severely prolonged ED stays, while multidisciplinary consultation significantly reduced risk.

**Conclusion:**

Severely prolonged ED stays among older adults are driven by both patient-level vulnerabilities and system-level constraints. Early nutritional screening, timely infection management, and multidisciplinary collaboration may help reduce boarding time and optimize care for aging populations.

## 1. Introduction

With the global acceleration of population aging, emergency department (ED) is witnessing a growing influx of older adults. According to the World Health Organization, individuals aged 65 and above account for a substantial proportion of healthcare utilization, particularly in emergency services [1]. Within this context, prolonged emergency department stays (PEDs) among geriatric patients have emerged as a critical concern, posing challenges to both patient outcomes and ED operational efficiency [2–4].

A growing body of literature has demonstrated that older adults tend to experience longer ED stays compared with their younger counterparts [5, 6]. This can be attributed to geriatric-specific factors such as multimorbidity, cognitive impairment, functional dependence, and diagnostic complexity [4]. PEDs have been associated with adverse outcomes, including higher risks of in-hospital complications, increased admission rates, longer inpatient stays, and elevated mortality, while also negatively affecting patient satisfaction and care experience [7–9].

Importantly, recent systematic evidence has examined the impact of extended ED length of stay (LOS) on patient outcomes. Burgess et al. conducted a comprehensive systematic review and reported that PEDs were frequently, though not uniformly, associated with poorer outcomes, such as higher risks of adverse events, treatment delays, and increased mortality [2]. These findings suggest that while the clinical and systemic consequences of PEDs are increasingly recognized, the magnitude and consistency of their effects vary across patient populations and healthcare contexts, underscoring the need for timely and targeted interventions [3].

While extensive research has been conducted on ED crowding and patient throughput [10], studies specifically examining geriatric PEDs remain limited, especially across different healthcare systems. Moreover, definitions of prolonged ED LOS vary internationally, which affects comparability and generalizability of results. For example, in many Western healthcare systems, boarding is defined as any ED stay exceeding 4–6 h [11–13], whereas in China, the threshold for PEDs is typically set at 72 h. Such discrepancies underscore the importance of contextualized and localized evidence when interpreting findings and developing targeted strategies.

This study, therefore, seeks to address these gaps by examining the condition and clinical characteristics of PEDs among geriatric patients in a Chinese tertiary hospital and identifying key determinants contributing to extended ED LOS. Findings from this study are expected to inform evidence-based strategies to optimize ED care delivery and improve health outcomes for the aging population.

## 2. Method

### 2.1. Design

This study was designed as a single-center, retrospective cohort study based on routinely collected electronic health records.

### 2.2. Sample and Setting

The study population consisted of all adult patients who experienced prolonged ED stays at a tertiary general hospital in Hangzhou, Zhejiang Province, China. Data were extracted from the hospital's emergency information system for the period between January 1, 2024, and December 31, 2024. Inclusion criteria were (1) age ≥ 18 years and (2) total ED stay exceeding 72 h. No exclusions were made based on sex, diagnosis, or disposition. For further analysis, patients were stratified into geriatric (≥ 60 years) and nongeriatric groups to compare demographic characteristics, clinical features, and outcomes.

### 2.3. Data Collection

The study included all geriatric patients who experienced prolonged ED stays, defined as remaining in the ED observation unit for more than 72 h. This threshold was adopted in accordance with the national standard issued by the National Health Commission of China [14], which designates 72 h as the maximum permissible ED observation period. In addition, ED stays exceeding 96 h were classified as severely prolonged for subgroup analyses.

The primary outcome was ED LOS, calculated as the interval between the time of entry into the ED observation unit and the time of departure, whether through discharge, transfer, or admission. A comprehensive set of variables was collected to characterize patients and explore potential determinants of prolonged ED stays. Demographic and socioeconomic variables included age, sex, marital status (married, single, widowed, and divorced), and type of medical insurance (urban employee insurance, rural cooperative medical care, self-payment, or other). Clinical urgency at arrival was evaluated using the standardized four-level triage system widely implemented in Chinese EDs, ranging from Level I (*most urgent*) to Level IV (*least urgent*).

Arrival time was categorized as daytime (08:00–19:59) or nighttime (20:00–07:59), based on the registration time in the ED. Multimorbidity was defined as the presence of two or more chronic diseases [15], as documented in the electronic medical records. Consciousness status at arrival was assessed using the Alert, Verbal, Pain, Unresponsive (AVPU) scale [16], while nutritional risk was determined using the Nutritional Risk Screening 2002 (NRS-2002) [17], with a score of ≥ 3 indicating significant risk. Additional variables included presence of infection (based on documented clinical diagnoses supported by laboratory or imaging evidence), whether a multidisciplinary consultation (MDC) was conducted (defined as involvement of two or more specialties), and whether a hospital bed was formally requested during the ED stay.

All collected data were de-identified to ensure patient confidentiality and comply with ethical research standards.

### 2.4. Analysis

Descriptive statistics were used to summarize patient demographics and clinical characteristics. Categorical variables were presented as frequencies and percentages, while continuous variables were reported as means with standard deviations or medians with interquartile ranges [*M* (Q1, Q3)], as appropriate. The analytical approach comprised three main steps. First, to compare the geriatric and nongeriatric cohorts, we used chi-square tests for categorical variables and Student's *t*-test or Mann–Whitney *U* test for continuous variables. Second, within the geriatric cohort, we performed univariate logistic regression analyses to examine the crude associations between various factors and a severely prolonged ED stay. Third, variables significant in the univariate analysis (*p* < 0.05) were entered into a multivariable logistic regression model to identify independent determinants. The Hosmer–Lemeshow test was used to assess the model's goodness of fit, and model performance was evaluated by accuracy (the proportion of correctly classified cases).

Statistical significance was defined as *p* < 0.05. All analyses were performed using IBM SPSS Statistics Version 21.0.

### 2.5. Ethical Approval

The study was approved by the Ethics Committee of the tertiary general hospital in Hangzhou (Approval no: IIT20250880B-R1). Given the retrospective nature of the study and the use of anonymized data, informed consent was waived. All procedures complied with the Personal Information Protection Law of the People's Republic of China and institutional data governance regulations.

## 3. Result

A total of 652 patients who experienced prolonged ED stays were included in the analysis. All patients included in this study were nontraumatic cases, and none were declared dead in the ED. More than 60% of the patients had two or more final diagnoses, reflecting a high level of clinical complexity. Regarding patient disposition, 5% were admitted to the intensive care unit (ICU), 15% were discharged against medical advice, and the remaining patients were admitted to inpatient wards. These findings emphasize the predominance of medically complex, nontraumatic cases and the diverse pathways of care following prolonged ED stays. No cases were lost to follow-up or excluded due to missing data. All ED boarding patients applied for inpatient beds within 24 h of observation.

### 3.1. Demographic and Clinical Characteristics of Geriatric and Nongeriatric ED Boarding Patients

Compared with nongeriatric patients, geriatric patients were older by definition and demonstrated a higher burden of multimorbidity and nutritional risk. They were also less likely to present during nighttime hours. Insurance type and the need for multidisciplinary consultation differed significantly between groups, while sex, marital status, triage level, and consciousness status showed no notable differences (Table 1).

### 3.2. Factors Associated With Severely Prolonged ED Stay in Geriatric Patients

Univariate regression identified several variables significantly associated with severely prolonged ED stays among geriatric patients. Advanced age, nighttime arrival, multimorbidity, nutritional risk, and infection were risk factors, while multidisciplinary consultation appeared protective. Sex, marital status, insurance type, triage level, and consciousness status showed no significant associations (Table 2).

In multivariate analysis, advanced age, nighttime arrival, nutritional risk, and infection remained independent predictors of severely prolonged ED stays in geriatric patients. Conversely, multidisciplinary consultation significantly reduced the likelihood of extended boarding. This highlights the interplay of patient-related vulnerabilities and systemic care processes in shaping ED LOS (Table 3).

## 4. Discussion

This study investigated determinants of severely prolonged ED stays among geriatric patients in a large tertiary hospital in China. Our findings highlighted several independent predictors, including advanced age, nighttime arrival, nutritional risk, and infection, while multidisciplinary consultation emerged as a protective factor. Beyond patient-level factors, structural characteristics of the health system also played a pivotal role.

### 4.1. Age and Nighttime Arrival

Advanced age was independently associated with severely PEDs. This finding is consistent with international evidence showing that older patients tend to experience longer ED boarding due to multimorbidity, frailty, and diagnostic complexity [6, 18, 19]. In our cohort, this effect was amplified for patients arriving at night. Night arrivals often face constrained resources, including reduced staffing levels and limited diagnostic services, which prolong throughput [20].

In addition, our study revealed that nighttime geriatric patients were frequently of very advanced age, often originating from nursing homes or living alone. The absence of immediate family members to provide history or consent further delayed diagnostic and therapeutic decisions. Cultural and personal preferences also contributed: many elderly patients and families favored conservative treatment and preferred prolonged observation in ED over inpatient admission once acute symptoms were stabilized.

Previous studies have reported mixed findings on the effect of arrival time. While some confirm nighttime as a risk factor for longer ED stays [21], others find no consistent association [22], often depending on local staffing models and boarding definitions. Our study suggests that in contexts of extreme boarding, the interaction of age and nighttime arrival exerts a particularly strong influence. Future research should further explore system-level modifiers, such as staffing patterns and diagnostic turnaround times, to clarify causal pathways.

In interpreting these findings, some caution is needed. The night arrival variable may act as a proxy for unmeasured factors (e.g., disease severity, delays in recognition, and lower priority in triage during off hours) [23]. Also, reverse causality is possible: patients who already require more time may tend to arrive later (e.g., due to delays in care-seeking) [24]. Future studies should control for staffing levels, diagnostic turnaround times, and disease acuity stratification to better isolate the pure effect of night arrival on prolonged ED stay.

### 4.2. Nutritional Risk as a Key Predictor

In this study, nutritional risk was a strong independent predictor of severely PEDs among geriatric patients. This aligns with broader evidence in hospitalized populations [25], where poor nutritional status is associated with prolonged inpatient LOS, higher complication rates, and delayed recovery [26]. Although fewer studies have focused specifically on nutritional risk in the ED setting [27], our findings reinforce the clinical importance of early nutritional assessment in the emergency context.

From a mechanistic perspective, elderly patients at nutritional risk often have diminished physiological reserves [26], impaired immunity [28], and poorer wound healing [29], which may prompt a more cautious and gradual diagnostic and therapeutic approach during their ED stay. Clinicians may delay aggressive interventions until baseline nutritional issues are addressed or stabilized, thereby extending observation periods [27]. In addition, patients with nutritional risk tend to have more comorbidities and organ dysfunction, compounding the time required for interventions and monitoring [30].

Our results are consistent with those from inpatient settings: for instance, Mendes et al. reported that nutritional risk on admission correlated significantly with prolonged hospital LOS in older patients with COVID-19 [31]. In the ED setting, Duman et al. also found that malnutrition risk among ED patients was nontrivial and positively associated with age [32]. However, because nutritional status is often under-assessed or under-documented in ED workflows, its role in ED boarding has been underappreciated.

Contrastingly, some ED-focused studies of LOS do not consider nutrition or include it only as a covariate with weak effect sizes [27]. This may reflect measurement limitations, heterogeneity in screening tools, or low priority in urgent care contexts [33]. The fact that nutritional risk retains significance in a model of extreme ED boarding underscores both the robustness of its effect and the clinical need to integrate nutritional screening into emergency workflows [34].

One limitation is that nutritional risk may correlate with unmeasured severity or comorbidity burden, thus serving as a surrogate marker. We attempted to adjust for multimorbidity and infection, but residual confounding may remain. Future prospective work should incorporate standardized nutritional assessment tools (e.g., NRS-2002) at ED arrival and monitor dynamic nutritional changes during the stay to clarify causality.

### 4.3. Infection and Prolonged Stay

In our multivariate model, the presence of infection emerged as an independent risk factor for severely PEDs in geriatric patients. This is biologically plausible and supported by external evidence [35]. Infection often triggers a cascade of diagnostic and therapeutic processes, including serial laboratory testing, cultures, imaging, initiation of antimicrobial therapy, monitoring of response, and possible escalation or consultation [36, 37]. In geriatric patients with compromised physiological reserve, hemodynamic instability or fluctuating vital signs may require more cautious observation, which prolongs stay [38].

In the ED literature, sepsis or suspected infection is frequently associated with prolonged boarding and adverse outcomes. For example, Yealy et al. emphasized that ED boarding poses additional risk for patients with suspected sepsis, where timely interventions are critical [39]. More broadly, extended ED stays in infected patients may increase the risk of nosocomial complications, delirium, or deterioration [40]. Conversely, if an ED is overwhelmed, infection cases may receive delayed diagnostics or management, further increasing time in ED [36].

However, some prior ED LOS studies do not consistently find infection as an independent predictor, likely because infection is often subsumed under “acuity” or “disease severity” covariates, which dilute its unique effect [41]. In our context of extreme boarding, the effect of infection may become more visible. Nonetheless, one must consider that infection status could be a proxy for severity or comorbidity burden; we attempted to adjust for multimorbidity, but residual confounding remains possible.

A limitation in our study is that we defined “infection” broadly based on chart documentation and laboratory results. We could neither uniformly grade infection severity nor fully capture timing of antimicrobial initiation. Future research would benefit from distinguishing infection subtypes, severity indices, temporal dynamics of diagnostic workup, and linkage to ED to ward transition delays to further refine the relationship.

### 4.4. Multidisciplinary Consultation as a Protective Factor

One of the more encouraging findings in our study was that multidisciplinary consultation was independently associated with a lower risk of severely prolonged ED stay in geriatric patients. This suggests that coordinated, cross-specialty input may expedite decision-making, streamline care pathways, and reduce unnecessary delays in diagnostic or therapeutic planning [42–44].

The benefit of multidisciplinary consultation in complex patients is well recognized in inpatient and specialized care settings, though less studied in the ED boarding context [45]. In theoretical terms, having relevant specialists engaged early may facilitate parallel rather than sequential evaluation, reducing redundant investigations and preventing waiting time caused by “back and forth” between departments. It may also enable more efficient consensus on disposition and reduce indecision delays.

Some prior studies on ED throughput advocate integrated care teams to reduce LOS, though few isolate the effect in prolonged boarding scenarios. For example, Adams et al. argue in a public health perspective that adopting multidisciplinary coordination helps mitigate boarding delays, particularly when hospital capacity is strained [46]. However, actual empirical evidence quantifying this effect is limited. Our finding provides real-world support in a challenging high-delay environment.

There remain caveats. The association might be influenced by selection bias: patients who receive multidisciplinary consultation may already be identified as high complexity or priority cases and thus cared for under more intensive oversight. It is also possible that institutions that are more capable of offering multidisciplinary input have generally better operational resources (staffing and bed management), confounding the association. Furthermore, our study does not directly measure the timing or efficiency of consultations. Future work should explore the “dose” and timeliness of consultation, perhaps via time to consult metrics, to delineate optimal consultation strategies for reducing boarding durations.

### 4.5. Structural Determinants of ED Boarding

Beyond patient-level risk factors, structural and system-level determinants strongly influence ED boarding durations, particularly in contexts where a definition of “prolonged stay,” such as 72 h, reflects institutional constraints. In China, the 72-h threshold often serves as a regulatory boundary for ED observation units; exceeding it implies systemic capacity stress rather than purely patient-specific delay. As such, prolonged stays may reflect hospital bed scarcity, inefficient bed assignment systems, and suboptimal inpatient throughput rather than individualized clinical inertia [13, 47].

Indeed, the literature indicates that boarding is a systemic phenomenon: ED crowding, delayed bed turnover, mismatched capacity planning, and suboptimal patient flow models all contribute to prolonged ED LOS [3, 48]. Some authors propose mitigation strategies such as establishment of bed management command centers, dynamic bed allocation algorithms, early discharge planning, and cross-department coordination to reduce boarding burden [49].

In our study setting, because all patients already crossed the 72-h threshold, further variation in boarding duration may be heavily influenced by hospital-level policies, such as prioritization of admissions, scheduling of diagnostics and care teams, and capacity mobilization during peak demand. For example, when inpatient wards are saturated, even clinically ready patients may linger in ED. Moreover, time-of-day effects and shift-based resource constraints may amplify delays for night arrivals, creating a feedback loop where structure exacerbates patient-level risk [50].

Some critiques argue that without structural reform, interventions targeting individual-level predictors have limited ceiling effects [48]. That is, even if nutrition, infection, or consultation is optimized, if bed allocation and throughput remain bottlenecks, ED boarding will persist. Our findings support a dual‐pronged response: while improving clinical-level efficiency is important, structural investment in bed capacity, process redesign, and hospital-wide flow management are indispensable [51, 52].

### 4.6. Limitations

As a single-center retrospective study, variables such as social support, functional status, and home care were not captured, potentially underestimating nonmedical influences on boarding. Furthermore, the study focused exclusively on boarding patients; future research including nonboarding cases would strengthen comparative insights.

## 5. Conclusions

In summary, our findings demonstrate that both patient-specific vulnerabilities and systemic constraints contribute to PEDs among geriatric patients. Advanced age, nighttime arrival, nutritional risk, and infection significantly increase risk, while multidisciplinary consultation offers protective value. However, structural bottlenecks in hospital capacity remain central drivers of PEDs. Addressing this multifactorial issue will require both individualized care strategies and system-wide reforms to improve patient flow and outcomes.

## Figures and Tables

**Table 1:** Comparison of demographic and clinical characteristics between geriatric and nongeriatric ED boarding patients.

Variable		Total (*n* = 652)	Nongeriatric (*n* = 174)	Geriatric (*n* = 478)	*Z*/*χ*^2^	*p* value
ED length of stay (hrs), *M* (Q1, Q3)		99.9 (85.4, 140.8)	99.4 (86.0, 141.5)	100.4 (84.1, 140.5)	−0.046	0.96

Age (years), *M* (Q1, Q3)		68 (58, 75)	51 (39.8, 55)	72 (67, 78)	−19.555	< 0.001^∗^

Sex, *n* (%)	Male	379 (58.1)	95 (54.6)	284 (59.4)	1.216	0.27
	Female	273 (41.9)	79 (45.4)	194 (40.6)		

Marital status, *n* (%)	Married	317 (48.6)	89 (51.1)	228 (47.7)	0.608	0.44
	Single/Divorced/Widowed	335 (51.4)	85 (48.9)	250 (52.3)		

Type of insurance, *n* (%)	Urban employee insurance	214 (32.8)	56 (32.2)	158 (33.1)	16.374	< 0.001^∗^
	Resident insurance	398 (61.0)	118 (67.8)	280 (58.6)		
	Self-pay/Other	40 (6.1)	0 (0)	40 (8.4)		

Triage level, *n* (%)	Level I	154 (23.6)	41 (23.6)	113 (23.6)	1.125	0.57
	Level II	176 (27.0)	42 (24.1)	134 (28.0)		
	Level III	322 (49.4)	91 (52.3)	231 (48.3)		

Arrival time, *n* (%)	Daytime (08:00–19:59)	464 (71.2)	110 (63.2)	354 (74.1)	7.305	0.007^∗^
	Nighttime (20:00–07:59)	188 (28.8)	64 (36.8)	124 (25.9)		

Multimorbidity, *n* (%)	Yes	347 (53.2)	64 (36.8)	283 (59.2)	25.763	< 0.001^∗^
	No	305 (46.8)	110 (63.2)	195 (40.8)		

Consciousness level, *n* (%)	Alert	327 (50.2)	88 (50.6)	239 (50.0)	0.017	0.90
	Altered	325 (49.8)	86 (49.4)	239 (50.0)		

Nutritional risk, *n* (%)	Yes^a^	333 (51.1)	63 (36.2)	270 (56.5)	20.992	< 0.001^∗^
	No	319 (48.9)	111 (63.8)	208 (43.5)		

Infection, *n* (%)	Present	341 (52.3)	81 (46.6)	260 (54.4)	3.144	0.08
	Absent	311 (47.7)	93 (53.4)	218 (45.6)		

Multidisciplinary consultation, *n* (%)	Yes	372 (65.0)	61 (35.1)	311 (65.1)	46.873	< 0.001^∗^
	No	280 (35.0)	113 (64.9)	167 (34.9)		

Severely prolonged stay, *n* (%)	Yes^b^	376 (57.7)	98 (56.3)	278 (58.2)	0.176	0.68
	No	276 (42.3)	76 (43.7)	200 (41.8)		

*Note: Z* = Mann–Whitney *U* test, *χ*^2^ = Chi-square.

^a^NRS-2002 ≥ 3. ^b^Prolonged stay > 24 h.

^∗^ *p* < 0.05.

**Table 2:** Univariate logistic regression analysis of severely prolonged ED stay among geriatric patients (*n* = 478).

Variable		*p* value	OR (95% CI)
Age		< 0.001^∗^	1.063 (1.039–1.088)

Sex		0.97	0.994 (0.686–1.439)

Marital status		0.63	1.049 (0.760–1.574)

Insurance type	Urban employee insurance^R^		
	Resident insurance	0.28	0.664 (0.314–1.402)
	Self-pay/Other	0.08	0.524 (0.256–1.072)

Triage level	Level I^R^		
	Level II	0.17	1.384 (0.873–2.196)
	Level III	0.38	1.213 (0.788–1.868)

Arrival time^a^		0.01^∗^	1.732 (1.126–2.665)

Multimorbidity		0.02^∗^	1.554 (1.074–2.250)

Consciousness status		0.58	0.902 (0.627–1.297)

Nutritional risk		< 0.001^∗^	2.691 (1.850–3.916)

Infection		< 0.001^∗^	2.065 (1.427–2.988)

Multidisciplinary consultation		< 0.001^∗^	0.079 (0.046–0.137)

^a^Nighttime.

^R^Reference.

^∗^ *p* < 0.05.

**Table 3:** Multivariate regression analysis of determinants of severely prolonged ED stay in geriatric patients^a^ (*n* = 478).

Variable	*β* coefficient	SE	Wald	*p* value	OR	95% CI
Age	0.030	0.014	4.331	0.04^∗^	1.030	1.002–1.059
Arrival time	0.668	0.255	6.885	0.009^∗^	1.950	1.184–3.211
Nutritional risk	0.816	0.226	13.018	< 0.001^∗^	2.262	1.452–3.524
Infection	0.530	0.221	5.759	0.02^∗^	1.699	1.102–2.619
Multidisciplinary consultation	−2.397	0.291	67.712	< 0.001^∗^	0.091	0.051–0.161

^a^The chi-square statistic was 11.87 (*p*=0.16), suggesting no statistical significance at *α* = 0.05 level. The model achieved 74.1% classification accuracy.

^∗^ *p* < 0.05.

## Data Availability

The datasets generated and analyzed during the current study are not publicly available due to institutional data protection policies but are available from the corresponding author upon reasonable request.
